# Age-Dependent Susceptibility to Enteropathogenic *Escherichia coli* (EPEC) Infection in Mice

**DOI:** 10.1371/journal.ppat.1005616

**Published:** 2016-05-09

**Authors:** Aline Dupont, Felix Sommer, Kaiyi Zhang, Urska Repnik, Marijana Basic, André Bleich, Mark Kühnel, Fredrik Bäckhed, Yael Litvak, Marcus Fulde, Ilan Rosenshine, Mathias W. Hornef

**Affiliations:** 1 Institute for Medical Microbiology, RWTH Aachen University Hospital, Aachen, Germany; 2 Institute of Medical Microbiology and Hospital Epidemiology, Hannover Medical School, Hannover, Germany; 3 The Wallenberg Laboratory, Department of Molecular and Clinical Medicine, University of Gothenburg, Gothenburg, Sweden; 4 Department of Biosciences, University of Oslo, Oslo, Norway; 5 Institute for Laboratory Animal Science, Hannover Medical School, Hannover, Germany; 6 Institute for Functional and Applied Anatomy, Hannover Medical School, Hannover, Germany; 7 Department for Microbiology and Molecular Genetics, Institute of Medical Research Israel-Canada, Faculty of Medicine, The Hebrew University of Jerusalem, Jerusalem, Israel; University of Michigan Medical School, UNITED STATES

## Abstract

Enteropathogenic *Escherichia coli* (EPEC) represents a major causative agent of infant diarrhea associated with significant morbidity and mortality in developing countries. Although studied extensively *in vitro*, the investigation of the host-pathogen interaction *in vivo* has been hampered by the lack of a suitable small animal model. Using RT-PCR and global transcriptome analysis, high throughput 16S rDNA sequencing as well as immunofluorescence and electron microscopy, we characterize the EPEC-host interaction following oral challenge of newborn mice. Spontaneous colonization of the small intestine and colon of neonate mice that lasted until weaning was observed. Intimate attachment to the epithelial plasma membrane and microcolony formation were visualized only in the presence of a functional bundle forming pili (BFP) and type III secretion system (T3SS). Similarly, a T3SS-dependent EPEC-induced innate immune response, mediated *via* MyD88, TLR5 and TLR9 led to the induction of a distinct set of genes in infected intestinal epithelial cells. Infection-induced alterations of the microbiota composition remained restricted to the postnatal period. Although EPEC colonized the adult intestine in the absence of a competing microbiota, no microcolonies were observed at the small intestinal epithelium. Here, we introduce the first suitable mouse infection model and describe an age-dependent, virulence factor-dependent attachment of EPEC to enterocytes *in vivo*.

## Author Summary

Enteropathogenic *Escherichia coli* (EPEC) is an important causative agent of infant diarrhea associated with significant morbidity and mortality particularly in the developing world. Current knowledge on EPEC pathogenesis has mainly emanated from *in vitro* studies as research is limited by the absence of a suitable small animal infection model. Here, we use neonate mice and present a new infection model that mimics the hallmark of the EPEC–host cell interaction in humans. We observe microcolonies of EPEC closely attached to the epithelial surface in the infected small intestine dependent on the presence of two well-established bacterial virulence factors, namely the type III secretion system and bundle forming pili. Studying the mucosal host response, we demonstrate enhanced epithelial expression of a distinct set of genes as well as an alteration of the intestinal microbiota composition. In contrast, EPEC fails to induce similar changes in adult animals illustrating the age-dependent susceptibility to EPEC infection. In the future, the new model could help to better understand the underlying mechanisms of EPEC infection and lead to the development of new therapeutic strategies to improve the outcome of infection in children.

## Introduction

Gastrointestinal infections remain a major cause of morbidity and mortality in the pediatric population worldwide. Among them, infections with enteropathogenic *Escherichia coli* (EPEC) have been recognized to exhibit a great pathogen-attributable risk of death in infants aged 0–11 months [1]. Insight into the interaction between EPEC and the host has mostly been derived from *in vitro* studies using immortalized cell lines. These studies demonstrated that type IV bundle forming pili (BFP) mediate the initial contact between EPEC and the host epithelial cell and are responsible for the typical localized adherence pattern observed at the epithelial surface [2–4]. The bacterium-cell interaction is further strengthened by the translocation of the translocated intimin receptor (Tir) *via* the type III secretion system (T3SS), resulting in the formation of typical attaching and effacing (A/E) lesions [5]. Additional effector molecules translocated by the T3SS were shown to induce massive cytoskeletal reorganization, manipulate host cell signaling and induce epithelial apoptosis [6–9].

In the past, the lack of a suitable small animal model has prevented a detailed analysis of the host-microbial interactions during infection *in vivo* [10]. EPEC infections have already been examined in larger animals such as rabbits, pigs or calves [9,11]. These models, however, are not amenable to genetic modifications and germ-free animals are not widely available. In addition, *Citrobacter rodentium*, a natural mouse enteropathogen, is used as a model organism for EPEC infection. However, although *C*. *rodentium* shares many features with EPEC, their tissue tropism, histopathology and clinical symptoms after infection differ. Therefore *in vivo* epithelial host responses to EPEC, protective antimicrobial host factors as well as the influence of the enteric microbiota and the consequences of EPEC infection on host-microbial homeostasis have all remained ill-defined.

Here we present the establishment of a new oral model of EPEC infection using neonate mice. Oral administration induced effective intestinal colonization. Bacterial attachment to the epithelial apical surface *in vivo* was associated with the generation of A/E lesion-like focal microcolonies dependent on the presence of functional BFP and T3SS. Transcriptome and RT-PCR analysis of wildtype and gene-deficient animals illustrated the epithelial response to EPEC infection and identified the innate immune receptors involved. High throughput 16S rDNA sequencing revealed infection-induced alterations of the developing microbiota. Finally, microcolony formation was shown to be restricted to the neonatal period despite efficient colonization of adult animals in the absence of a competitive enteric microbiota. Thus, we present a new oral EPEC infection model and demonstrate the age-restricted development of typical features associated with EPEC infection.

## Results

### Efficient colonization of the neonate intestinal tract by EPEC

**Fig 1:**
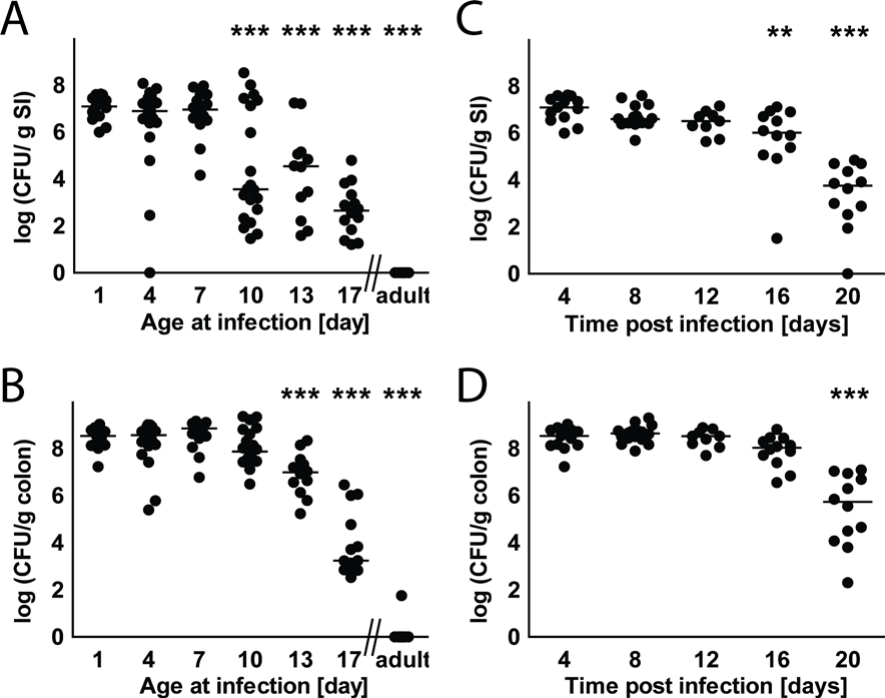
Intestinal colonization following oral administration. **(A-B)** Neonate and adult mice were orally infected with WT EPEC. Small intestine **(A)** and colon **(B)** tissues were collected 4 days p.i., homogenized and plated on LB agar plates supplemented with the appropriate antibiotic (n = 12-23/time point from at least 2 litters, median). **(C-D)** 1-day-old mice were orally infected with WT EPEC. Small intestine **(C)** and colon **(D)** tissues were collected at defined time points p.i., homogenized and plated on LB agar plates supplemented with the appropriate antibiotic (n = 9-16/time point from at least 3 litters; median). ANOVA with Dunnett’s post-test. **, p<0.01; ***, p<0.001.

Initially, 0.5 to 1x10^5^ CFU EPEC (strain E2348/69) were orally administered to mice at different ages and bacterial colonization was monitored at 4 days post infection (p.i.). Animals infected during their first week of life exhibited efficient intestinal colonization with high bacterial numbers recovered from small intestinal and colon tissue. Significantly lower numbers of colonizing bacteria were noted in the small and large intestine of animals infected after the age of 10 and 13 days, respectively (**Fig 1A and 1B**). Oral infection of adult animals even with high bacterial number (0.5 to 1x10^8^ CFU) did not lead to detectable colonization (**Fig 1A and 1B**). Subsequently, we analyzed the duration of the colonization following oral administration to 1-day-old neonates. Efficient colonization was noted during the first 12 days following bacterial challenge (**Fig 1C and 1D**). Colonization along the length of the small intestine took place primarily in the distal part (**S1A Fig**). A significant reduction in bacterial colonization was first observed at day 16 p.i. and day 20 p.i. in the small intestine and colon, respectively. Despite the high intestinal burden, no bacterial spread to systemic organs occurred (**S1B and S1C Fig**). Also, no measurable increase in intestinal permeability, clinical signs of disease, such as diarrhea, weight loss or mortality were observed in wild type mice (**S1D, S1E and S1F Fig**). These results demonstrate that EPEC efficiently colonizes the intestinal tract of mice during the first two weeks of life but fails to persist in the intestine of adult animals.

### Generation of epithelium-associated EPEC microcolonies

**Fig 2:**
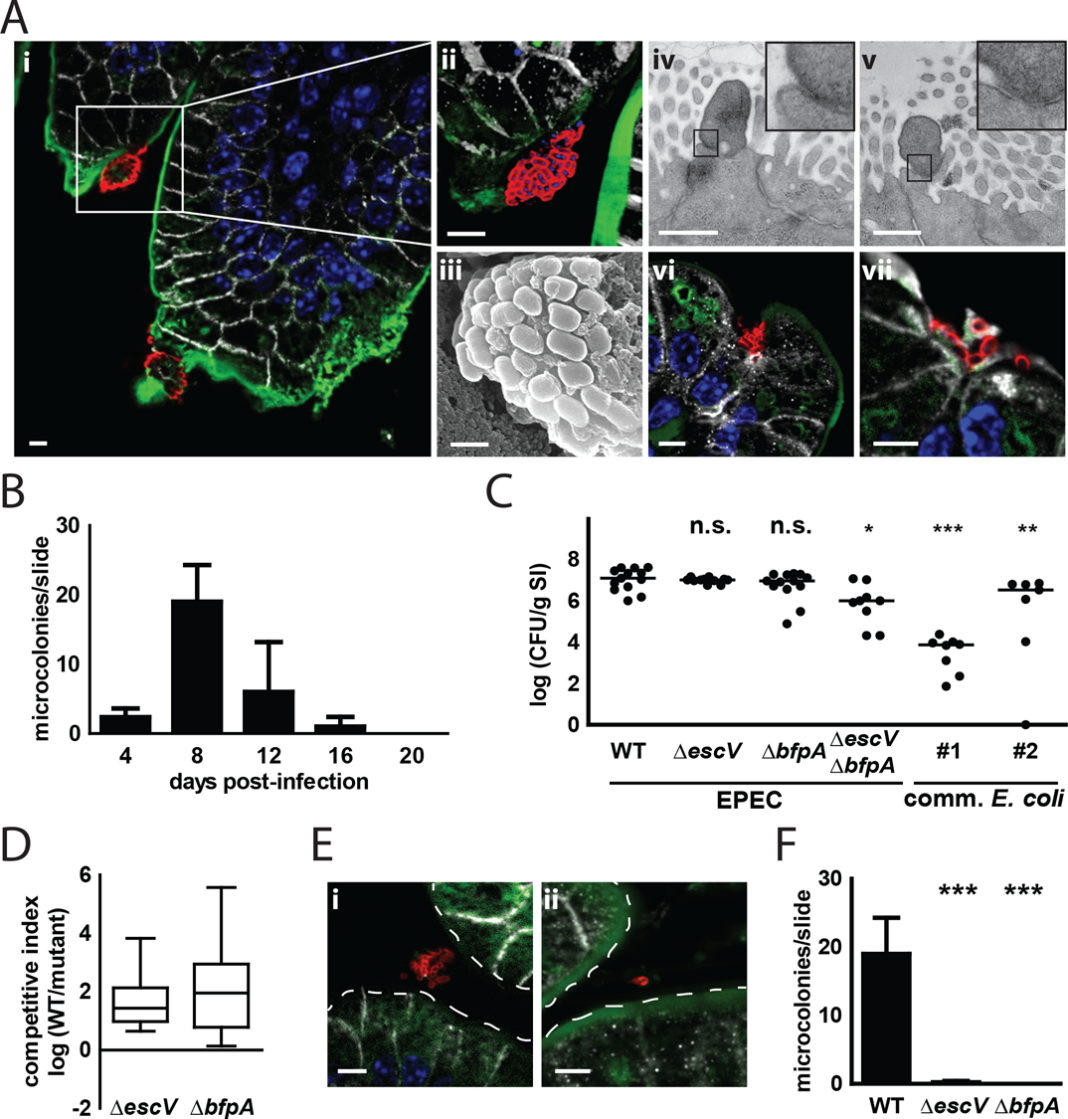
T3SS- and BFP-dependent generation of epithelial microcolonies *in vivo*. **(A)** Immunostaining and electron microscopy of small intestinal tissue sections collected 8 days p.i. from mice orally infected on the day of birth with WT EPEC. **(Ai;vi-vii)** 2d representation of microcolonies attached to the small intestinal epithelium. **(Aii)** enlarged 3d representation of the insert marked in **(Ai)** (EPEC, red; E-cadherin, white; wheat germ agglutinin (mucus), green; DAPI, blue, bar = 5μm). **(Aiii)** SEM of EPEC forming a microcolony (bar = 1 μm). **(Aiv-v)** TEM of EPEC attached to the epithelial plasma membrane (bar = 0.5 μm). **(B)** Number of microcolonies per small intestinal tissue section at defined time points p.i. (n = 9 from 3 mice per time point, mean ± SD). **(C)** 1-day-old mice were orally exposed to WT EPEC, *escV* or *bfpA* single mutants, a *escV*/*bfpA* double mutant or two commensal *E*. *coli* strains (#1, a murine commensal *E*. *coli* isolate; #2, *E*. *coli* Nissle). Small intestinal tissues were collected 4 days p.i., homogenized and plated on LB agar plates supplemented with streptomycin (WT), kanamycin (*escV*, *bfpA*, *escV*/*bfpA* mutants) or ampicillin (commensal *E*. *coli strains*) (n = 7–13 from at least 2 litters; median). **(D)** 1-day-old mice were orally co-infected with WT EPEC and either *escV* or *bfpA* mutants at a 1:1 ratio (total: 1–2×10^5^ CFU). Total small intestinal tissues were collected 8 days p.i., homogenized and plated on different LB agar plates supplemented with the appropriate antibiotic to discriminate WT EPEC from *escV* or *bfpA* mutants (n = 15–24 from at least 2 litters; data are represented in box and whisker plot format). **(E)** Immunostaining of small intestinal tissue sections collected 8 days p.i. from mice orally infected at birth with *escV* **(i)** or *bfpA* mutants **(ii)** (EPEC, red; E-cadherin, white; wheat germ agglutinin (mucus), green; DAPI, blue; bar = 5μm). **(F)** Number of microcolonies observed per small intestinal tissue section in animals infected with WT EPEC or with *escV* or *bfpA* mutants at 8 days p.i. (n = 9 from 3 mice per time point, mean ± SD). ANOVA with Dunnett’s post-test (C and F). ns, p>0.05; *, p<0.05; **, p<0.01; ***, p<0.001.

Next, we visualized the interaction of EPEC with the small intestinal epithelium. Immunostaining and scanning electron microscopy (SEM) revealed the formation of typical bacterial microcolonies composed of densely packed bacteria attached to the epithelial surface (**Fig 2A_i_–2A_iii_**). Transmission electron microscopy (TEM) illustrated the tight contact between the bacterial surface and the apical plasma membrane of the epithelium (**Fig 2A_iv_ and 2A_v_**). The tight attachment of EPEC to the epithelial plasma membrane was further illustrated by the crescent-shaped staining pattern observed on attached bacteria by fluorescence immunostaining for bacterial lipopolysaccharide (**Fig 2A_vii_**). Of note, microcolonies were preferentially localized at uncharacterized epithelial asperities or folds of the epithelial surface (**Fig 2A_vi_ and 2A_vii_**). This might explain the difficulties to visualize microcolonies by electron microscopy. Microcolonies were observed as early as 4 days p.i., and their number peaked at 8 days p.i. Fewer microcolonies were observed at later time points and were absent from tissue sections obtained at 20 days p.i. (**Fig 2B**). No EPEC microcolonies could be observed in colonic tissue (**S2A Fig**).

To investigate the involvement of the two major EPEC pathogenicity factors BFP and T3SS during intestinal colonization and microcolony formation, mutants deficient in the biogenesis of BFP (Δ*bfpA*), T3SS (Δ*escV*) or both (Δ*bfp*,*A* Δ*escV*) and two commensal *E*. *coli* strains were analyzed (**S2B Fig**). EPEC *bfpA* and *escV* single mutants as well as the *bfpA*/*escV* double mutant and the two commensal *E*. *coli* strains readily colonized the neonate small intestine and colon (**Fig 2C and S2C Fig**). Some variation in the colonization efficacy was noted. In particular, the EPEC *escV*/*bfpA* double mutant and both commensal *E*. *coli* strains exhibited a somewhat reduced colonization efficacy. In contrast, EPEC *bfpA* and *escV* single mutants displayed a colonization efficacy similar to WT EPEC. In a direct comparative analysis, however, WT EPEC bacteria outcompeted both, *bfpA* or *escV*, mutants when administered simultaneously at a 1:1 ratio to neonates (**Fig 2D and S2D Fig**). Importantly, *bfpA* and *escV* EPEC mutants completely failed to generate microcolonies at the small intestinal epithelial surface at 8 days p.i. (**Fig 2E and 2F**). Thus, BFP and the T3SS are critical determinants of microcolony formation but only marginally contribute to colonization of the neonate intestine.

### EPEC-mediated innate immune stimulation of the intestinal epithelium

**Fig 3:**
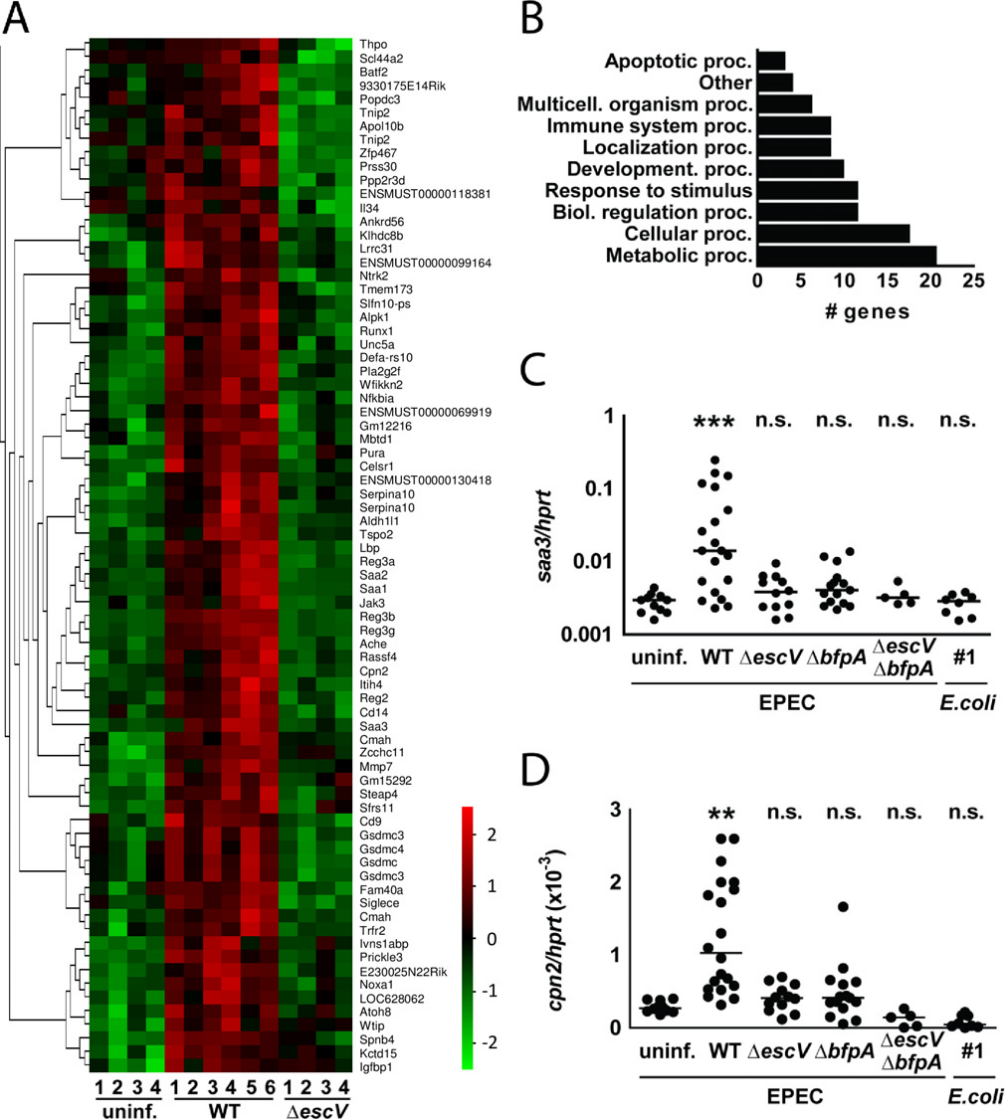
Characterization of the epithelial response to EPEC infection *in vivo*. **(A)** Heat map showing the intestinal epithelial gene expression in small intestinal tissue of uninfected mice (uninf.) as well as at day 8 p.i. with WT EPEC or *escV* mutant (n = 4–6 from at least 2 litters). A selection of the most significantly up-regulated genes following WT EPEC infection are shown (p-value = 0.02; q-value = 0.22). **(B)** Clusters of orthologous group (COG) analysis of the genes shown in **(A)**. **(C-D)** 1-day-old mice were orally infected with WT EPEC, *escV* mutant, *bfpA* mutant, *escV*/*bfpA* double mutant EPEC, a murine commensal *E*. *coli* strain or left untreated. IEC were isolated from the small intestinal tissue at 8 days p.i. and the expression levels of **(C)** *saa3* and **(D)** *cpn2* were determined by quantitative RT-PCR and normalized to the values obtained for the housekeeping gene *hprt* (n = 4–16 from at least 2 litters; median). Kruskal-Wallis ANOVA with Dunn’s multiple comparison post-test (C-D). ns, p>0.05; **, p<0.01; ***, p<0.001.

Subsequently, we analyzed the epithelial transcriptional response to EPEC exposure. Global transcriptome analysis using total mRNA prepared from isolated primary intestinal epithelial cells (IEC) revealed a defined set of genes that were upregulated after infection (**Fig 3A**). Remarkably, transcriptional stimulation of these genes was completely absent following infection with the T3SS-deficient (Δ*escV*) mutant indicating the requirement of an intact T3SS for epithelial stimulation. Genes induced after EPEC infection were mostly involved in metabolic, cellular and regulatory processes as well as responses to exogenous stimulation (**Fig 3B**). Quantitative RT-PCR for the acute phase reactant serum amyloid A3 (*saa3)* and the carboxypeptidase N (*cpn2)* confirmed the EPEC-induced epithelial cell response and additionally revealed the strong influence of BFP expression for epithelial stimulation (**Fig 3C and 3D**). These results suggest a functional link between microcolony formation and the induction of epithelial gene expression.

**Fig 4:**
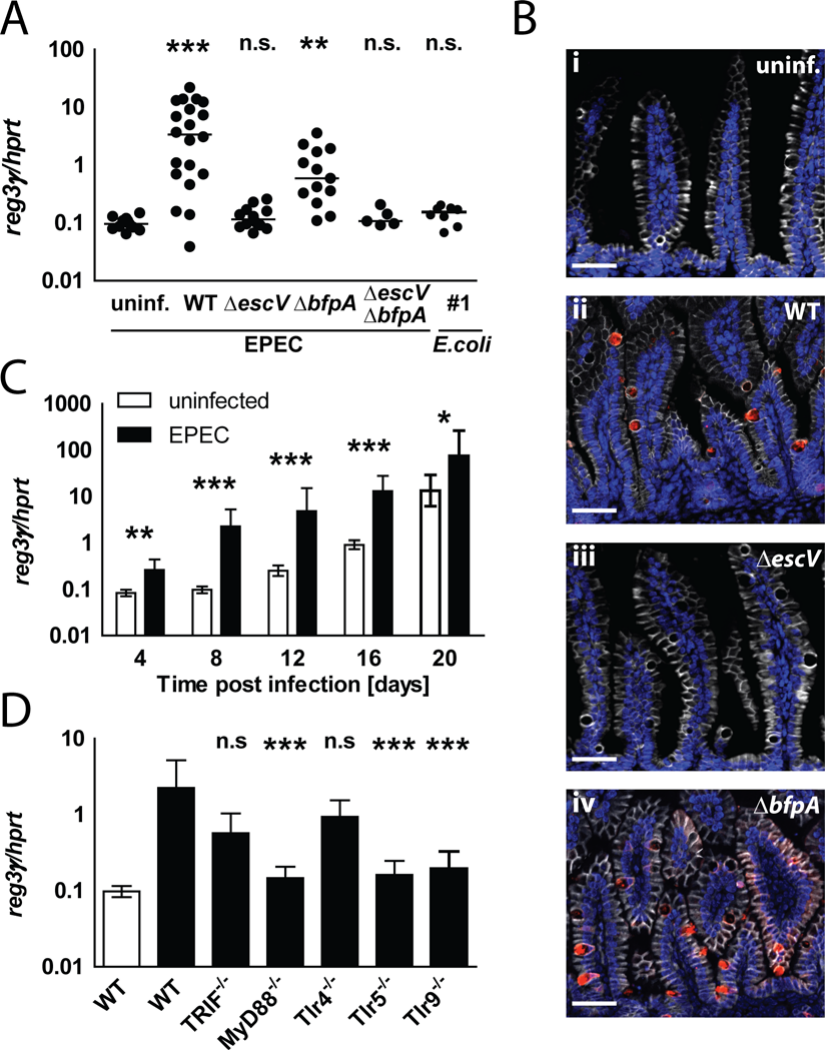
Analysis of bacterial and host factors required for the EPEC-induced epithelial response. **(A)** 1-day-old mice were orally infected with WT EPEC, *escV* mutant, *bfpA* mutant, *escV*/*bfpA* double mutant EPEC, a murine commensal *E*. *coli* strain or left untreated. IEC were isolated 8 days p.i. and the expression level of *RegIIIγ* was determined and normalized to the values obtained for *hprt* (n = 4–16 from at least 2 litters; median). **(B)** Immunostaining for RegIIIγ in the distal part of the small intestine of 9-day-old untreated control animals **(i)** or mice infected at birth with WT **(ii)**, *escV* mutant **(iii)**, or *bfpA* mutant EPEC **(iv)** at 8 days p.i. (RegIIIγ, red; E-cadherin, white; DAPI, blue; bar = 50 μm). **(C)** Time kinetic of the epithelial *RegIIIγ* expression in infected neonates and age-matched controls. 1-day-old mice were orally infected with WT EPEC (black bars) or left untreated (white bars). IEC were isolated at 5, 9, 13, 17 and 21 days after birth and the expression of *RegIIIγ* was measured and normalized to *hprt* (n = 7–17 from at least 2 litters; geometric mean ± 95% confidence interval). **(D)** 1-day-old C57BL/6 WT, MyD88^-/-^, TRIF^-/-^, Tlr4^-/-^, Tlr5^-/-^ and Tlr9^-/-^ mice were orally infected with WT EPEC (black bars) or left untreated (white bars) and the epithelial *RegIIIγ* expression was measured and normalized to *hprt* (n = 11–27 from at least 2 litters; geometric mean ± 95% confidence interval). Kruskal-Wallis ANOVA with Dunn’s multiple comparison post-test (A and C-D). ns, p>0.05; *, p<0.05; **, p<0.01; ***, p<0.001.

Next, we examined the expression of the antibacterial c-type lectin *RegIIIγ*, one of the most highly (65-fold) upregulated genes 8 days p.i., in more detail. Whereas EPEC-induced epithelial expression of *RegIIIγ* was completely abolished in the absence of a functional T3SS, a significant, albeit reduced, epithelial innate immune stimulation was observed following infection with BFP-deficient (Δ*bfpA*) EPEC (**Fig 4A**). This finding was corroborated by immunostaining. RegIIIγ positive goblet cells were only observed in the distal part of the small intestine of animals infected with WT EPEC and EPEC *bfpA* mutants (**Fig 4B**). Of note, basal epithelial *RegIIIγ* expression increases with age after birth [12]. Direct comparison with age-matched control animals, however, demonstrated a significantly enhanced epithelial *RegIIIγ* expression following challenge (**Fig 4C**). To characterize the upstream signaling events of the EPEC-mediated innate immune stimulation, WT mice were compared with animals deficient in innate immune receptors known to mediate epithelial innate immune recognition. A significant reduction in epithelial *RegIIIγ* expression was noted in the absence of MyD88, Tlr5 or Tlr9 (**Fig 4D**). In addition, impaired innate host responses enhanced the susceptibility to EPEC infection, demonstrating the critical role played by these molecules in the mucosal response to EPEC infection (**S1F Fig**).

### EPEC-induced alteration of the developing enteric microbiota

**Fig 5:**
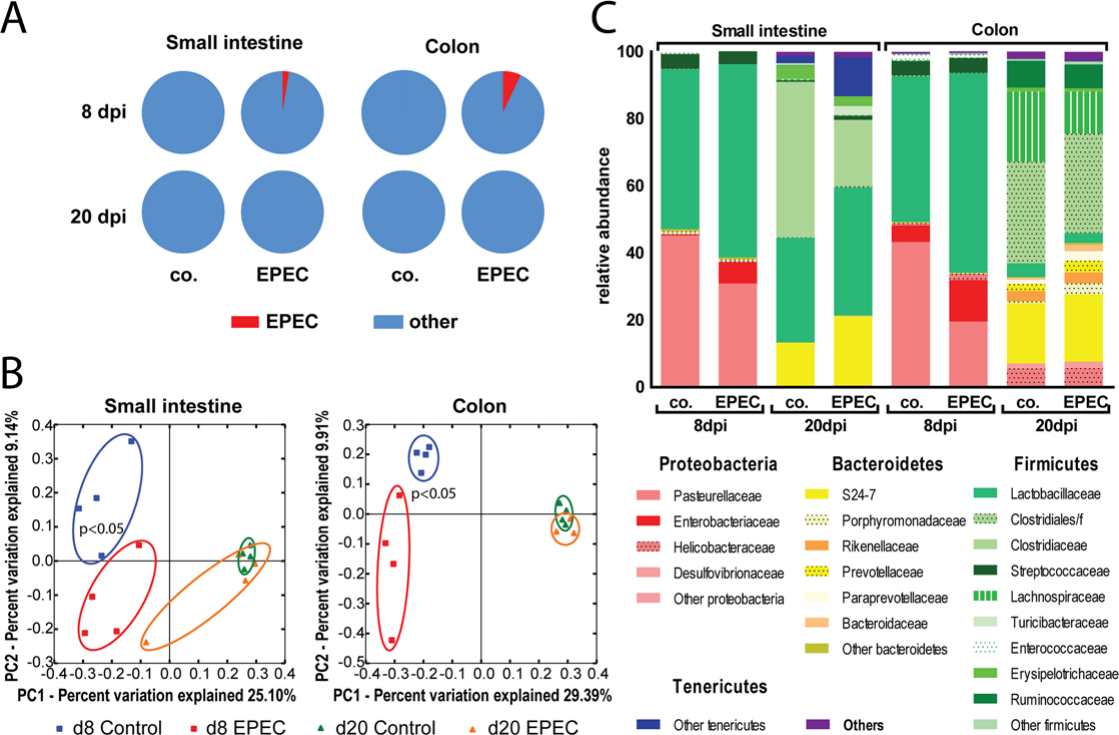
EPEC infection temporally alters the composition of the enteric microbiota. Bacterial DNA was extracted from the small intestine and colon of newborn mice infected with WT EPEC at 8 and 20 days p.i. or untreated age-matched control animals and analyzed by 16S rDNA sequencing. **(A)** Relative abundance of the OTU 4425571 (Enterobacteriaceae, representing EPEC in red) in infected and uninfected animals. **(B)** PC1/PC2 plot illustrating the similarity of the microbiota within the different groups in the small intestine (left) and in the colon (right) (d8 uninfected, blue squares; d8 EPEC-infected, red squares; d20 uninfected, green triangles; d20 EPEC-infected, orange triangles). **(C)** Phylum level composition of the 8 different groups. Proteobacteria are represented in different shades of red, Bacteroides in different shades of yellow, Firmicutes in different shades of green and Tenericutes in blue.

In addition, we carefully analyzed the composition of the enteric microbiota in the small intestine and colon of infected and non-infected animals 8 and 20 days p.i. by 16S rDNA sequencing. At 8 days p.i., at the height of the infection, EPEC (OTU 4425571) accounted for 2.6% or 7.1% of the total bacteria present in the small intestine or colon, respectively. At 20 days p.i., when the infection was being resolved, EPEC could not be detected anymore in either the small intestine or the colon (**Figs 1C, 1D and 5A**). PCA plot analysis revealed a significant influence of EPEC on the intestinal bacterial community at 8 days p.i. In fact, the microbiota in the small intestine and colon differed between infected (red squares) and non-infected control (blue squares) animals at day 8 p.i. (p-value = 0.0181 for small intestine and 0.0147 for colon [PC2]) but was superimposable in both organs after recovery at day 20 p.i. (green triangles *versus* orange triangles) (p-value = 0.2006 for small intestine and 0.3341 for colon [PC2]) (**Fig 5B**). A highly significant age-dependent difference in the microbiota composition was noted, with a decrease in Proteobacteria and a concomitant rise of bacteria of the obligate anaerobic phylum Bacteroidetes between 9- and 21-day-old animals. This is consistent with major changes in nutrients (breast milk *vs* solid food), the local luminal milieu and the establishment of an increasingly diverse enteric microbiota. The overall phylum composition between the infected samples and their uninfected age-matched controls was, however, remarkably similar for both the small intestine and the colon (**Fig 5C**). Significant differences in the bacterial composition between infected and control animals were detected at 8 days p.i. and these differences were also observed after removal of the EPEC-related sequences from the analysis. Also, bacterial groups expanded concomitantly with the decrease in EPEC colonization but were absent in uninfected animals (**S1 Table**).

### Microbiota-dependent colonization but age-dependent microcolony formation and innate immune stimulation

**Fig 6:**
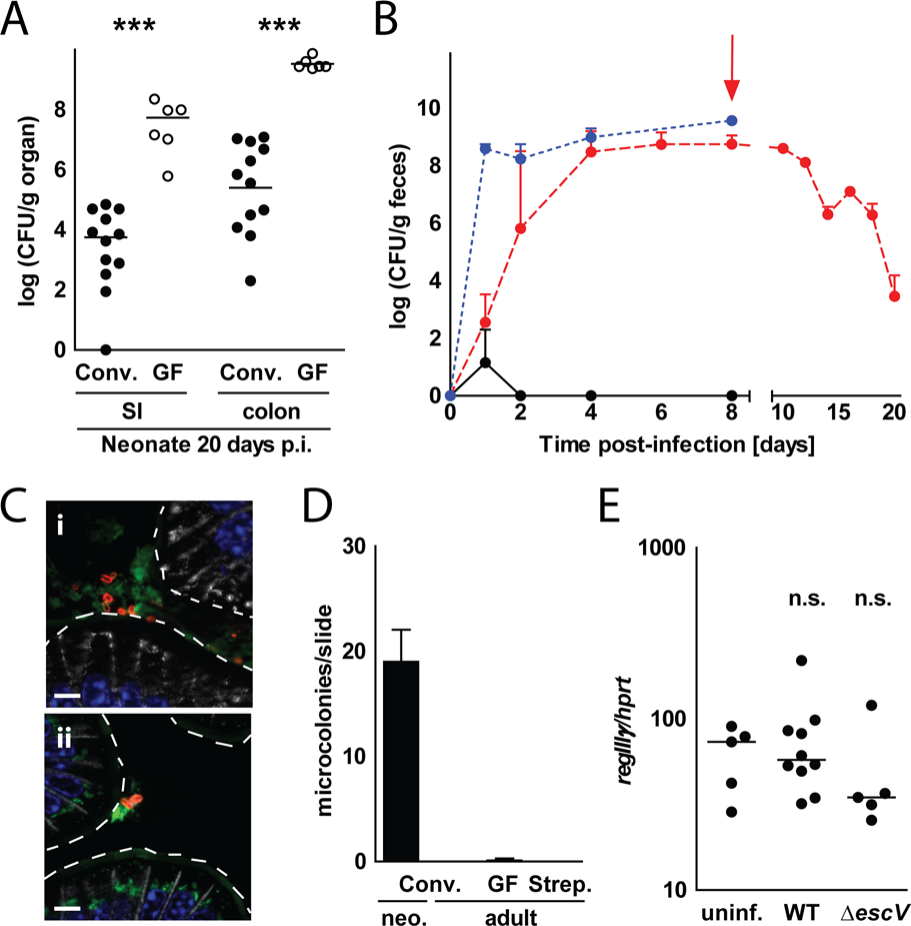
The influence of the enteric microbiota and intestinal development on colonization and microcolony formation. **(A)** 1-day-old conventional (conv.) and germ-free (GF) mice were orally infected with WT EPEC. Small intestinal and colon tissues were collected 20 days p.i., homogenized and plated on LB agar plates supplemented with the appropriate antibiotic (n = 6–12 from 1 litter for GF mice and 5 litters for conventional mice; median). **(B)** Conventional adult mice treated with streptomycin in drinking water (red dotted line) or left untreated (black line) as well as untreated adult germ-free mice (blue dotted line) were infected with WT EPEC. Streptomycin treatment was stopped 8 days p.i. (red arrow). Feces were collected at the indicated time point p.i., homogenized and plated on LB agar plates supplemented with the appropriate antibiotic (n = 3–33; median ± interquartile range). **(C)** Immunostaining of small intestinal tissue sections collected 8 days p.i. from adult streptomycin-treated **(i)** and germ-free mice **(ii)** orally infected with WT EPEC (EPEC, red; E-cadherin, white; wheat germ agglutinin (mucus), green; DAPI, blue; bar = 5μm). **(D)** Number of microcolonies observed per small intestinal tissue section of 1-day-old and non-treated, streptomycin-treated and GF adult animals infected with WT EPEC 8 days p.i. (n = 9 from 3 mice per time point, mean ± SD). **(E)** IEC were isolated from the small intestines of adult streptomycin-treated, WT EPEC or *escV* mutant-infected animals at 8 days p.i. and the expression level of *RegIIIγ* was measured and normalized to the values obtained for *hprt* (n = 5–10; median). Student’s t-test (A) and Kruskal-Wallis ANOVA with Dunn’s multiple comparison post-test (E). ns, p>0.05; ***, p<0.001.

The critical role of the emerging enteric microbiota in restricting EPEC colonization was demonstrated using germ-free (GF) mice. Conventional and GF mice were infected at day 1 after birth. Whereas the colonization in conventionally housed mice dropped significantly 20 days p.i., EPEC colonization in germ-free mice remained high with numbers similar to younger animals (**Fig 6A**). Additionally, we investigated the role of the enteric microbiota during infection of adult animals. As previously shown, conventionally raised adult mice were resistant to EPEC colonization/infection (**Figs 1A, 1B and 6B**). However, EPEC was recovered from the feces of infected gnotobiotic mice or of conventionally raised mice treated with antibiotics (**Fig 6B**). EPEC colonization required continuous antibiotic administration since a sharp decrease in EPEC colonization was observed upon termination of antibiotic treatment (**Fig 6B**). Importantly, despite shedding high numbers of EPEC at 8 days p.i., no microcolony formation was observed in the gut of adult GF and streptomycin-treated mice (**Fig 6C and 6D**). In contrast, neonate GF mice similar to conventionally raised newborn animals, displayed EPEC microcolonies 8 days p.i. (**S3 Fig**). Also, epithelial *RegIIIγ* expression remained low upon infection of GF and streptomycin-treated adult animals (**Fig 6E**). Together, these results illustrate the role of the enteric microbiota in colonization resistance. The generation of microcolonies, hallmark of the EPEC-host cell interaction, was however age-dependent but microbiota-independent.

## Discussion

Here we present the first small animal model amenable to genetic modifications to investigate EPEC infection *in vivo*. EPEC was originally described to cause outbreaks of diarrhea in pediatric wards in industrialized countries but nowadays remains a major health concern in developing countries, particularly for small children and HIV-infected infants [1,13–15]. Although general principles of EPEC pathogenesis have been unraveled using *in vitro* and large animal models, the emerging picture is still incomplete.

Oral infection of neonate mice led to the histological hallmark of EPEC infection in humans: an A/E lesion-like localized adherence pattern on the surface of the small intestinal epithelium. Two previously defined important virulence factors, BFP and T3SS, were critical for microcolony formation as well as mucosal innate immune stimulation. Infection of neonate mice, however, was not accompanied by clinical symptoms such as watery diarrhea observed in humans. The reason is currently not clear but might result from differences in the physiology and ability to develop diarrhea between mice and men or from the degree of the infection and the infection-induced inflammatory tissue response. Nevertheless, we believe that the T3SS and BFP-dependent generation of A/E lesion-like microcolonies and the epithelial stimulation as characteristic features of EPEC infection make this model a useful tool to investigate the underlying mechanisms of EPEC infection and for testing novel therapeutic strategies in the future.

Intestinal colonization also occurred in the absence of BFP or of a functional T3SS. Similarly, orally administered commensal *E*. *coli* strains were able to colonize the neonate gastrointestinal tract. Thus, the ability to colonize the neonatal intestine did not represent a virulence trait of EPEC but rather reflected the low colonization resistance in the murine neonate intestine also observed in other species [16,17]. Most likely, epithelium-attached bacteria accounted for only a minor fraction of the total number of intestinal EPEC bacteria. The presence of a subpopulation of virulent, disease-promoting bacteria has been confirmed during experimental *Citrobacter rodentium* and *Salmonella* infection [18,19]. Also in our model, competition experiments identified the enhanced colonization capacity of fully virulent T3SS and BFP-positive EPEC bacteria. The ability to firmly adhere to the intestinal epithelial surface might help to avoid shedding by continuous mucus secretion and intestinal peristalsis and allow enhanced proliferation [20]. Future studies need to further address bacterial virulence gene expression in respect to microcolony formation and enhanced colonization.

Similar to the situation in the neonate mouse intestine, EPEC was able to colonize germ-free adult mice or adult animals pretreated with antibiotics [21]. Colonization resistance thus protects adult animals from EPEC colonization. Differences in the enteric microbiota might thus explain reports describing EPEC colonization of the adult intestine [22]. Importantly, consistent with previous reports, infection of germ-free or pretreated adult mice failed to induce epithelial stimulation, intimate epithelial attachment and the generation of A/E lesion-like microcolonies [21,23,24]. Thus, the ability to colonize the intestine represents a prerequisite for infection but is not sufficient to generate the characteristic structural and functional features, microcolony formation and innate immune stimulation. These features seem to be restricted to the neonatal small intestine.

Many aspects of the innate and adaptive immune system such as the antimicrobial peptide repertoire, mucus secretion, epithelial-turn over, immune cell maturation as well as metabolic and anatomical features differ between the neonate and adult intestine and may account for the differential susceptibility to infection. Age-dependent susceptibility was also observed for other age-related enteric pathogens such as *E*. *coli*, *Salmonella* or rotavirus but the underlying mechanisms for this observation have not been fully elucidated [25–28]. Currently, the factor responsible for the age-dependent ability of EPEC to form microcolonies and stimulate the epithelium is unknown. A better understanding might, however, improve the clinical management of children suffering from infection with enteropathogenic microorganisms.

EPEC infection resulted in a significant upregulation of a distinct set of genes in the intestinal epithelium. Antimicrobial effectors, apolipoproteins and metabolic molecules accounted for the majority of these genes, thereby illustrating the antimicrobial and adaptive changes of the epithelium to the infectious challenge. The induced antimicrobial host response might contribute to the somewhat altered morphology of adhering EPEC bacteria illustrated by TEM. Upregulation of genes occurred in a T3SS-dependent manner, since the response was abolished in the absence of the essential inner membrane protein of the T3SS apparatus EscV. BFP-deficient EPEC mutants exhibited a strongly reduced stimulatory potential but still allowed for *RegIIIγ* upregulation. BFP-negative bacteria have a functional T3SS and are able to intimately attach to the intestinal epithelium but exhibit a lower binding efficacy and fail to form microcolonies [29,30]. The reduced host response induced by BFP-negative EPEC may explain the frequent isolation of so-called atypical EPEC strains that lack expression of BFP and constitute an emerging form of EPEC in humans [31–33].

Notably, our results confirm goblet cells as a major source of RegIIIγ in neonate mice, whereas enterocytes and Paneth cells were shown to be the main producers of RegIIIγ in adult animals [12,34]. Interestingly, effector gene expression was found distant to the site of microcolony formation, suggesting that horizontal epithelial communication *via* direct epithelial-epithelial cell interaction or *via* the secretion of soluble mediators occurred [35]. Consistent with previous reports, enhanced *RegIIIγ* mRNA expression required the TLR signaling adapter molecule MyD88 [36]. *RegIIIγ* expression was induced during the postnatal period, in parallel to the establishment of the enteric microbiota [12]. Our data additionally identify the upstream TLRs involved in EPEC-induced *RegIIIγ* expression *in vivo*, namely TLR5 and TLR9. However, since the comparative analyses were not performed using littermates, an influence of differences in the microbiota composition between the mouse strains used cannot be excluded. Intestinal epithelial cells express TLR5 and TLR9 and recognition of the flagellated EPEC by Tlr5 *in vitro* has already been described in the literature [37,38]. Also TLR9 was shown to protect from mucosal damage but its role during intestinal bacterial challenge has not been investigated [39]. Interestingly, both TLR5 and TLR9 have been proposed to exhibit a polarized expression and EPEC was shown to manipulate the localization of TLR5 in epithelial cells suggesting that it might manipulate the pro-inflammatory epithelial response [37–39]. Further *in vivo* analyses will be required to identify host and microbial factors contributing to EPEC pathogenesis.

Our results identified major changes in the microbiota composition of uninfected mice between 9 and 21 days after birth that could be responsible for the rise in colonization resistance observed during this period. For example, the small intestinal pathobionts, segmented filamentous bacteria [SFB (*Candidatus arthromitus*)] were only observed in samples from older mice, in accordance with previous reports [40]. Consistent with the ability of EPEC to colonize streptomycin-pretreated mice, SFB have been shown to be highly susceptible to streptomycin treatment [41]. Moreover SFB were less abundant in infected mice and colonization with SFB was shown to inhibit intestinal colonization by rabbit-specific EPEC in the rabbit model [42].

Other significant alterations of the microbiota composition were observed after EPEC infection. Infections associated with tissue destruction, immune stimulation or metabolic changes significantly influence nutritional and antimicrobial aspects of the enteric milieu and microbiota alterations might contribute to the pathogenesis of diarrheal disease [43,44]. Finally, infection-induced microbiota alterations might ultimately restrict pathogen colonization [45]. Together our findings highlight the potential influence of the enteric microbiota in EPEC colonization resistance, disease progression and pathogen elimination. Further investigations are, however, required to demonstrate causal relationships and elucidate the potential therapeutic value.

In conclusion, we present a new small animal model amenable to genetic modifications to investigate EPEC infection. We confirm the critical role of bacterial virulence factors, characterize the antimicrobial host response and identify the innate immune receptors stimulated by EPEC *in vivo*. Finally, we demonstrate the infection-induced transient alteration of the neonate enteric microbiota and reveal a critical role for age-dependent, but not microbiota-dependent factors during EPEC infection. Therefore, this model might help to unravel the mechanisms involved in the EPEC-host cell interaction and facilitate a much-needed improvement in the clinical management of infected children worldwide [46].

## Materials and Methods

### Mice

Mice were bred locally and held under specific pathogen-free or germ-free conditions at the Hannover Medical School animal facility. C57Bl/6N mice were purchased from Charles River laboratories, TLR4^-/-^ (B6.B10ScN-Tlr4^lps-del^/JthJ), TLR5^-/-^ (B6.129S1-Tlr5^tm1Flv^/J), TRIF^-/-^ (C57BL/6J-Ticam1^Lps2^/J) and MyD88^-/-^ (B6.129P2(SJL)-Myd88^tm1.1Defr^/J) mice were obtained from Jackson laboratories. TLR9^-/-^ (B6.129P2-Tlr9^tm1Aki^) mice were kindly provided by M. Brinkmann (Helmholz Center for Infection Biology, Braunschweig, Germany).

### Ethics statement

All animal experiments were performed in compliance with the German animal protection law (TierSchG) and were approved by the local animal welfare committee (approval 13/1256 of the Niedersachsische Landesamt für Verbraucherschutz und Lebensmittelsicherheit Oldenburg, Germany).

### Bacteria

WT EPEC E2348/69 (Streptomycin^R^), Δ*escV* EPEC (*escV*::miniTn10*kan*; Streptomycin^R^, Kanamycin^R^), Δ*bfpA* EPEC (*bfpA*::Tn*phoA*; Kanamycin^R^), Δ*escV*/*bfpA* EPEC (Kanamycin^R^, Chloramphenicol^R^), *E*. *coli* Nissle 1917 and a murine small intestinal *E*. *coli* isolate were grown in Luria Broth supplemented with the appropriate antibiotics [47–49]. Both commensal *E*. *coli* strains were transformed with a GFP expression plasmid (pGFP, Ampicillin^R^) to confer ampicillin resistance [27].

### Infection model

1-day-old mice were defined as animals born maximum 24 hours before the infection and presenting a milk spot indicating previous breast-feeding by the dam. 1-, 4-, 7-, 10-, 13- or 17-day-old mice were orally infected with approximately 0.5–1×10^5^ CFU bacteria in PBS (1μl). 5- to 8-week-old adult mice were gavaged with approximately 0.5–1×10^8^ bacteria in PBS (100μl). Streptomycin-treated adult mice were given orally 20μg streptomycin (SIGMA) in PBS (50μl) 24 hours before receiving EPEC and had access *ad libitum* to drinking water containing streptomycin (5g/L) for the first 8 days of the experiment. For competition experiments, bacteria were mixed in a 1:1 ratio and 1-day-old mice were co-infected with 0.5–1×10^5^ bacteria of each strain in PBS (2μl). 4, 8, 12, 16 or 20 days p.i., the small intestine, colon, spleen, liver and/or mesenteric lymph nodes were collected in PBS, homogenized, diluted and plated on LB agar plates containing the appropriate antibiotic.

### Scanning electron microscopy

Small intestines of WT EPEC infected neonates were collected 8 days p.i., longitudinally opened, flushed and fixed for 1 hour at room temperature in 200mM HEPES, pH 7.35 containing 4% formaldehyde and 0.1% glutaraldehyde. Samples were then dehydrated using increasing acetone series. Critical point drying was performed using a CPD030 critical point dryer (Balzers, Lichtenstein) following manufacturer instructions. SEM was performed using a QuantaSEM (FEI) in high vacuum mode.

### Transmission electron microscopy

For the ultrastructural analysis, the distal end of small intestines of WT EPEC infected neonates were dissected 8 days p.i. and fixed with 1% glutaraldehyde in 0.2M HEPES buffer, pH7.4 overnight. For embedding, the intestine was cut into 3mm long segments and these were cut open longitudinally. Samples were post-fixed with 1% osmium tetroxide and contrasted with 2% uranyl acetate, both for 1 hour. Dehydration was performed with a graded ethanol series (70-80-90-95-100%), followed by progressive infiltration with epoxy resin and polymerization overnight at 60°C. 70nm thin transverse sections were prepared using an ultramicrotome (Ultracut EM UCT-Leica Microsystems) and a diamond knife (Diatome), and contrasted with 0.2% lead citrate for 15s. Samples were analyzed with JEM1400 transmission electron microscope (JEOL). Images were recorded with TemCam-F216 (Tvips).

### Immunofluorescence staining

Paraffin-embedded small intestinal and colonic tissue sections were stained using a rabbit anti-OK127 anti-serum (Statens Serum Institute) to visualize EPEC or a rabbit anti-RegIIIγ anti-serum (gift from Lora Hooper, Southwestern Medical Center, Texas, USA) and a mouse anti-E cadherin (BD Biosciences) antibody and fluorescein-labelled wheat germ agglutinin (Vector labs). Secondary antibodies used in this study were purchased from Dianova. Slides were then mounted in DAPI-mounting medium (Vectashield) and pictures were taken with an ApoTome microscope connected to a digital camera (Zeiss). Microcolony quantification was done by assessing the number of “clusters of at least 5 EPEC bacteria attached to the small intestinal epithelium” (microcolonies) per tissue section of the full organ. 3 non-consecutive sections from 3 animals were analyzed by experimental condition.

### Primary cell isolation and gene expression analysis

IEC were isolated from the small intestine as previously described [28]. Total RNA was isolated using TRIZOL (Invitrogen) and cDNA synthesized using RevertAid (Fermentas). Quantitative real-time PCR were performed using Taqman gene expression assays (Life Technologies): *hprt* (Mm00446968_m1), *regIIIγ* (Mm01181783_g1), *saa3* (Mm00441203_m1) and *cpn2* (Mm01169716_m1).

Microarray analysis was performed using Whole Mouse Genome Oligo Microarray v2 (4x44k) (Agilent Technologies) following the SC_AgilentV5.7 protocol provided by the manufacturer. The heat map was generated using Qlucore Omics explorer (p-value = 0.02; q-value = 0.21). Cluster of orthologous groups’ analysis was performed using PANTHER (http://www.pantherdb.org/). Expression array data are available through GEO Series accession number GSE71685.

### Microbiota analysis

Litters of 1-day-old mice were orally infected with WT EPEC or left untreated. 8 or 20 days p.i., full small intestine and full colon were collected, pooled (6 animals per sample for 9-day-old; 3 animals for 21-day-old) and snap frozen in liquid nitrogen. Bacterial DNA was extracted and analyzed by 16S rDNA sequencing.

### *In vivo* intestinal permeability assay

Mice left untreated or orally infected at birth with EPEC were orally fed 1μg of FITC dextran (MW 3000–5000—SIGMA) 8 days p.i. 4 hours later, their blood was withdrawn and the fluorescence intensity in their serum measured using a fluorometer (Victor).

### *In vitro* EPEC infection of intestinal epithelial cells

Small intestinal epithelial m-IC_cl2_ cells were cultured as previously described [50]. WT EPEC and Δ*escV* mutant carrying a green fluorescent protein (GFP) expression plasmid were used for *in vitro* infection experiments. Cells were grown on 8 chamber slides (Lab-Tek) for 6 days and were then left untreated or infected with WT EPEC or *escV* mutant at a MOI of 1 for 3 hours. Slides were then fixed with 4% PFA and stained using the CytoPainter F-actin staining kit (Abcam). Slides were then mounted in DAPI-mounting medium (Vectashield) and pictures were taken with an ApoTome microscope connected to a digital camera (Zeiss).

### List of accession numbers/ID numbers (NCBI database)

*Escherichia coli* 0127:H6 E2348/69 complete genome, Accession: FM180568.1

*Escherichia coli* O127a:H6 bfpA gene for bundlin-2a, complete cds, strain: E2348/69, Accession: AB247923.1

Mus musculus toll-like receptor 4 (Tlr4), mRNA, Accession: BC029856.1

Mus musculus toll-like receptor 5 (Tlr5), mRNA, Accession: NM_016928.3

Mus musculus toll-like receptor 9 (Tlr9), mRNA, Accession: NM_031178.2

Mus musculus IL-1 receptor related protein MyD88 mRNA, Accession: U84409.1

Mus musculus mRNA for Trif, mRNA, Accession: AB025010.1

Mus musculus regenerating islet-derived 3 gamma (Reg3g), mRNA, Accession: NM_011260.1

## Supporting Information

S1 Fig Clinical consequences of EPEC infection. **(A)** 1-day-old mice were orally infected with WT EPEC. The small intestine was collected at 8 days p.i. and divided into 3 equal parts. Each part was homogenized and plated on LB agar plates supplemented with the appropriate antibiotic (n = 10 from 2 different litters; mean ± SD). **(B-C) **1-day-old mice were orally infected with WT EPEC. Spleen (Sp.), liver (Li.) **(B)** and mesenteric lymph nodes (MLN) **(C)** were collected at 8 days p.i., homogenized and plated on LB agar plates supplemented with the appropriate antibiotic (n = 8 from 2 different litters; the median is indicated). **(D)** 1-day-old mice were orally infected with WT EPEC (filled circles) or left uninfected (empty circles). The animals were fed 1μg of 4kDa FITC-dextran 8 days p.i. and the concentration of FITC-dextran in serum was measured (n = 8 from 2 different litters; the median is indicated). **(E)** 1-day-old mice were orally infected with WT EPEC (filled circles) or left uninfected (empty circles) and their body weight was recorded on a daily basis (n = 18 from 2 different litters; mean ± SD). **(F)** 1-day-old C57BL/6 WT (black) and MyD88^-/-^ (red) mice were orally infected with WT EPEC (solid lines) or *escV* mutant (dotted lines) bacteria and monitored daily (n = 8–20 from at least 2 different litters; Kaplan-Meier survival curve). Student’s t-test (D) and Log-rank Mantel-Cox test (F). ns, p>0.05; ***, p<0.001.
(TIF)

S2 Fig Characterization of the different EPEC strains used in this study. **(A)** Immunostaining of colonic tissue sections collected 8 days p.i. from mice orally infected at birth with WT EPEC (EPEC, red; E-cadherin, white; wheat germ agglutinin (mucus), green; DAPI, blue; bar = 10μm). **(B)** mIC_cl2_ cells grown for 6 days on 8 chamber slides were left untreated (**i**) or infected with WT EPEC (**ii**) or *escV* mutant (**iii**) at a MOI of 1 for 3 hours. (GFP-EPEC, green; F-actin, red; DAPI, blue; bar = 20μm). **(C)** 1-day-old mice were orally infected with WT EPEC, *escV* or *bfpA* EPEC single mutants, *escV*/*bfpA* EPEC double mutant or two commensal *E*. *coli* strains (#1: commensal mouse isolate; #2 *E*. *coli* Nissle). Colon tissues were collected 4 days p.i., homogenized and plated on LB agar plates supplemented with streptomycin (WT), kanamycin (*escV*, *bfpA*, *escV*/*bfpA* mutants) or ampicillin (commensal *E*. *coli strains*) (n = 7–13 from at least 2 litters; median). **(D)** 1-day-old mice were orally co-infected with WT EPEC and either *escV* or *bfpA* mutants at a 1:1 ratio (total: 1–2×10^5^ CFU). Colon tissues were collected 8 days p.i., homogenized and plated on different LB agar plates supplemented with the appropriate antibiotic to discriminate WT EPEC from *escV* or *bfpA* mutants (n = 15–24 from at least 2 litters; box and whisker plot format). ANOVA with Dunnett’s post-test (C). ns, p>0.05; ***, p<0.001.
(TIF)

S3 Fig Microbiota-independent generation of EPEC microcolonies in neonates. **(A)** Immunostaining of small intestinal tissue sections collected 8 days p.i. from GF mice orally infected on the day of birth with WT EPEC (EPEC, red; E-cadherin, white; wheat germ agglutinin (mucus), green; DAPI, blue, bar = 5μm). **(B)** Number of microcolonies per small intestinal tissue section at day 8 p.i. (n = 12 from 3 mice, mean ± SD).
(TIF)

S1 Table OTUs significantly altered between infected and non-infected animals. Bacterial DNA was extracted from the small intestine and the colon of newborn mice infected with WT EPEC or left untreated at 8 and 20 days p.i. and analyzed by 16S rDNA sequencing. The table lists all OTUs that were significantly altered upon infection.
(XLSX)
