# Supplementary figures and images for: Age-Dependent Susceptibility to Enteropathogenic Escherichia coli (EPEC) Infection in Mice
Source: PLoS Pathog. 2016 May 9;12(5):e1005616. doi: 10.1371/journal.ppat.1005616 (PMC4861285; doi:10.1371/journal.ppat.1005616)

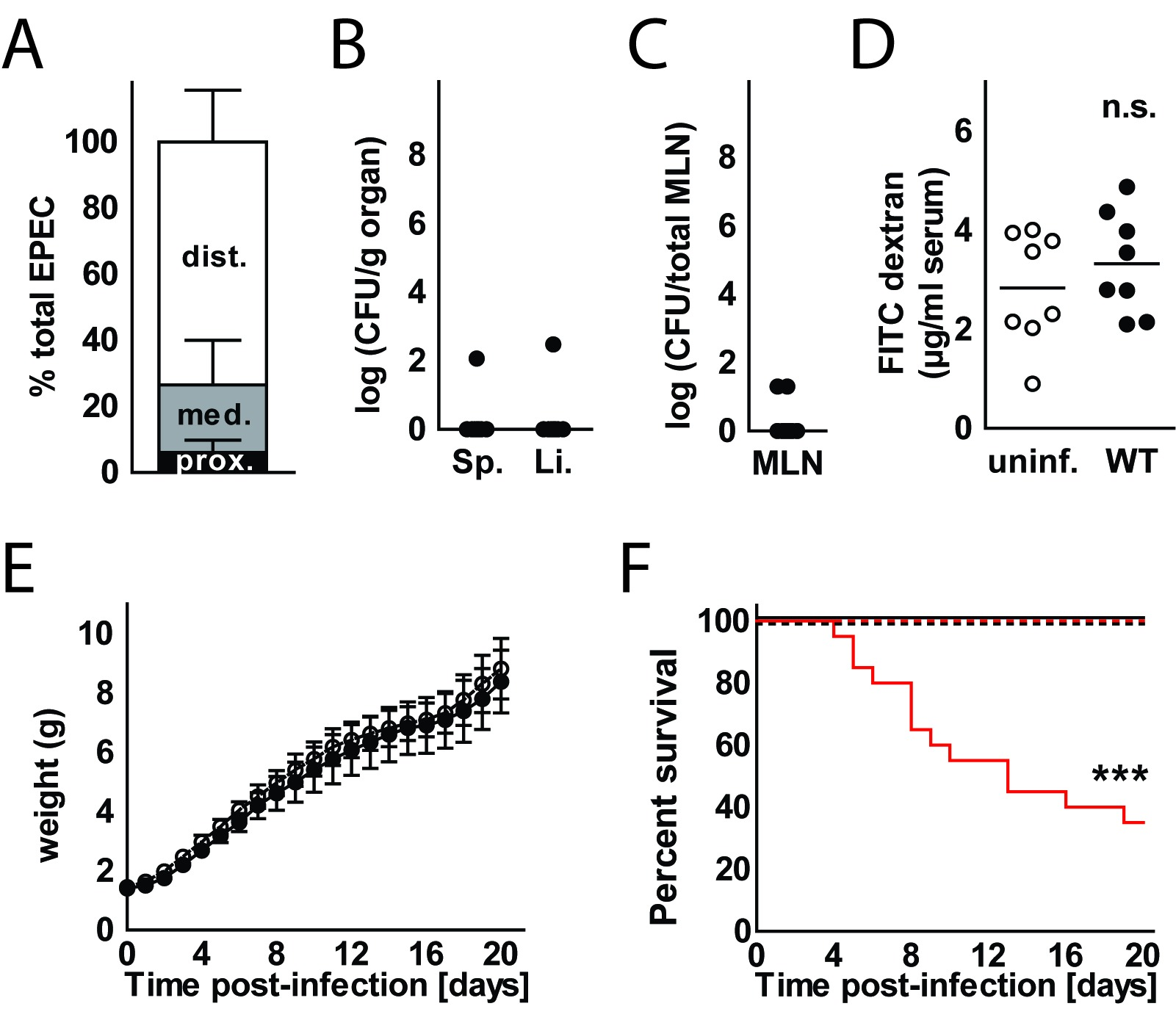

Supplement: S1 Fig — (A) 1-day-old mice were orally infected with WT EPEC. The small intestine was collected at 8 days p.i. and divided into 3 equal parts. Each part was homogenized and plated on LB agar plates supplemented with the appropriate antibiotic (n = 10 from 2 different litters; mean ± SD). (B-C) 1-day-old mice were orally infected with WT EPEC. Spleen (Sp.), liver (Li.) (B) and mesenteric lymph nodes (MLN) (C) were collected at 8 days p.i., homogenized and plated on LB agar plates supplemented with the appropriate antibiotic (n = 8 from 2 different litters; the median is indicated). (D) 1-day-old mice were orally infected with WT EPEC (filled circles) or left uninfected (empty circles). The animals were fed 1μg of 4kDa FITC-dextran 8 days p.i. and the concentration of FITC-dextran in serum was measured (n = 8 from 2 different litters; the median is indicated). (E) 1-day-old mice were orally infected with WT EPEC (filled circles) or left uninfected (empty circles) and their body weight was recorded on a daily basis (n = 18 from 2 different litters; mean ± SD). (F) 1-day-old C57BL/6 WT (black) and MyD88-/- (red) mice were orally infected with WT EPEC (solid lines) or escV mutant (dotted lines) bacteria and monitored daily (n = 8–20 from at least 2 different litters; Kaplan-Meier survival curve). Student’s t-test (D) and Log-rank Mantel-Cox test (F). ns, p>0.05; ***, p<0.001. (TIF) [file ppat.1005616.s001.tif]

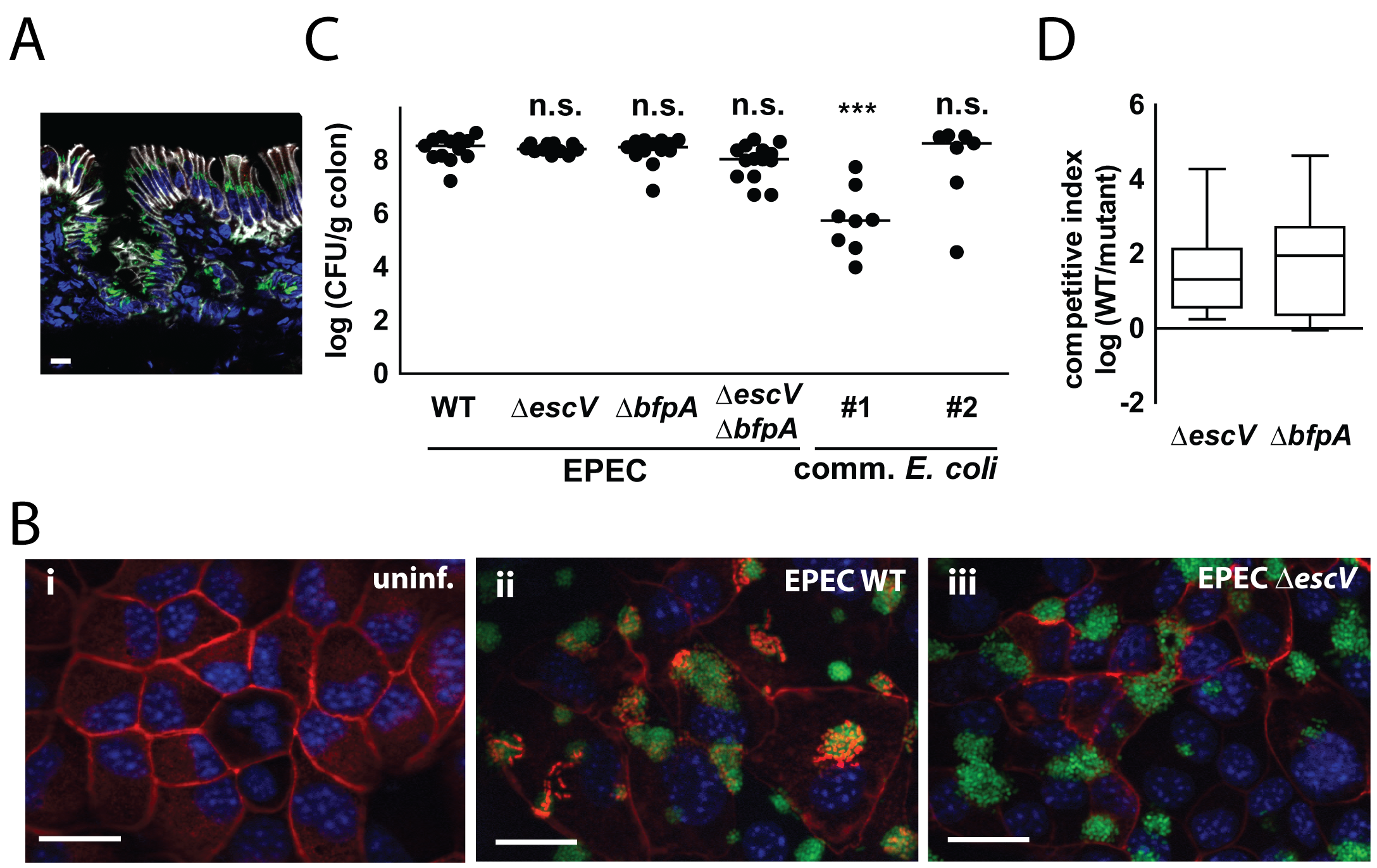

Supplement: S2 Fig — (A) Immunostaining of colonic tissue sections collected 8 days p.i. from mice orally infected at birth with WT EPEC (EPEC, red; E-cadherin, white; wheat germ agglutinin (mucus), green; DAPI, blue; bar = 10μm). (B) mICcl2 cells grown for 6 days on 8 chamber slides were left untreated (i) or infected with WT EPEC (ii) or escV mutant (iii) at a MOI of 1 for 3 hours. (GFP-EPEC, green; F-actin, red; DAPI, blue; bar = 20μm). (C) 1-day-old mice were orally infected with WT EPEC, escV or bfpA EPEC single mutants, escV/bfpA EPEC double mutant or two commensal E. coli strains (#1: commensal mouse isolate; #2 E. coli Nissle). Colon tissues were collected 4 days p.i., homogenized and plated on LB agar plates supplemented with streptomycin (WT), kanamycin (escV, bfpA, escV/bfpA mutants) or ampicillin (commensal E. coli strains) (n = 7–13 from at least 2 litters; median). (D) 1-day-old mice were orally co-infected with WT EPEC and either escV or bfpA mutants at a 1:1 ratio (total: 1–2×10^5^ CFU). Colon tissues were collected 8 days p.i., homogenized and plated on different LB agar plates supplemented with the appropriate antibiotic to discriminate WT EPEC from escV or bfpA mutants (n = 15–24 from at least 2 litters; box and whisker plot format). ANOVA with Dunnett’s post-test (C). ns, p>0.05; ***, p<0.001. (TIF) [file ppat.1005616.s002.tif]

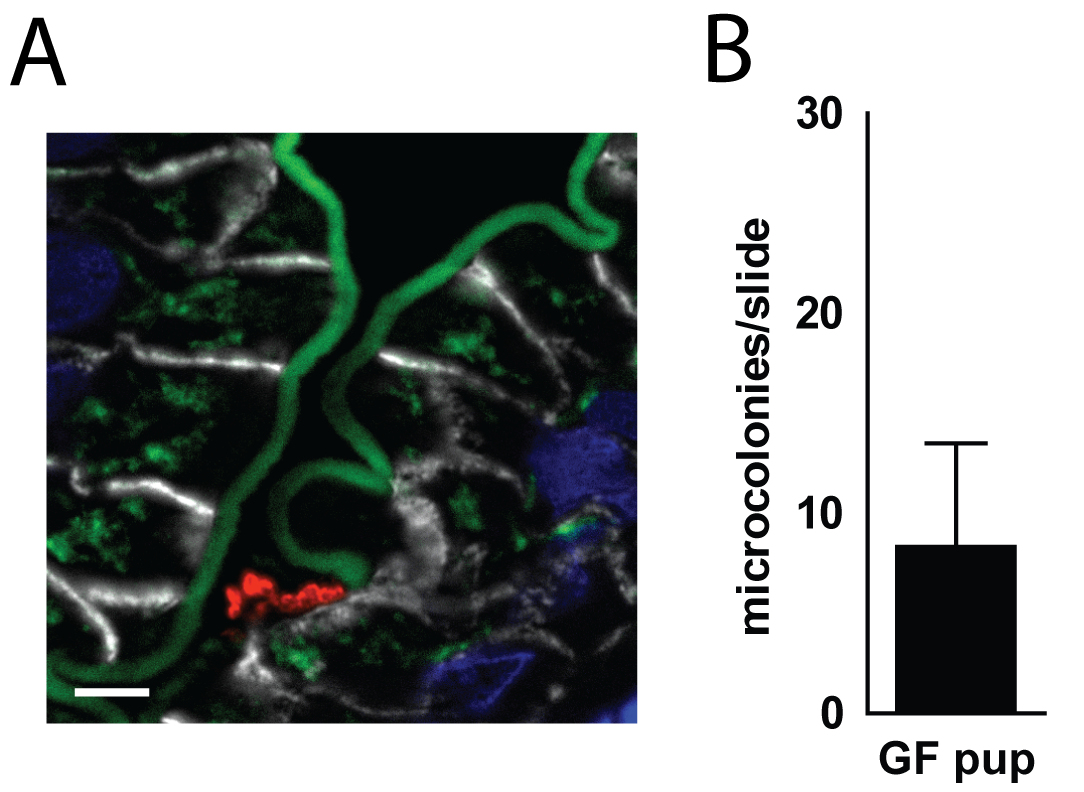

Supplement: S3 Fig — (A) Immunostaining of small intestinal tissue sections collected 8 days p.i. from GF mice orally infected on the day of birth with WT EPEC (EPEC, red; E-cadherin, white; wheat germ agglutinin (mucus), green; DAPI, blue, bar = 5μm). (B) Number of microcolonies per small intestinal tissue section at day 8 p.i. (n = 12 from 3 mice, mean ± SD). (TIF) [file ppat.1005616.s003.tif]
